# Early versus late surgery after cervical spinal cord injury: a Japanese nationwide trauma database study

**DOI:** 10.1186/s13018-019-1341-4

**Published:** 2019-09-05

**Authors:** Chie Tanaka, Takashi Tagami, Junya Kaneko, Reo Fukuda, Fumihiko Nakayama, Shin Sato, Akiko Takehara, Saori Kudo, Masamune Kuno, Masayoshi Kondo, Kyoko Unemoto

**Affiliations:** 1 Department of Emergency and Critical Care Medicine, Nippon Medical School Tama Nagayama Hospital, 1-7-1 Nagayama, Tama-shi, Tokyo, 2068512 Japan; 2 Department of Clinical Epidemiology and Health Economics, School of Public Health, The University of Tokyo, 7-3-1 Hongo, Bunkyo-ku, Tokyo, 1130033 Japan

**Keywords:** Cervical SCI, Mortality, Early surgery, Late surgery

## Abstract

**Background:**

The management of cervical spinal cord injury (SCI) has changed drastically in the last decades, and surgery is the primary treatment. However, the optimum timing of early surgical treatment (within 24 h or 72 h after injury) is still controversial. We sought to determine the optimum timing of surgery for cervical SCI, comparing the length of the intensive care unit (ICU) stay and in-hospital mortality in patients who underwent surgical treatments (decompression and stabilization) for cervical SCI within 24 h after injury and within 7 days after injury.

**Methods:**

This was a retrospective cohort study using Japan Trauma Data Bank (JTDB) which is a nationwide, multicenter database. We selected adult isolated cervical SCI patients who underwent operative management within 7 days after injury, between 2004 and 2015. The main outcome measures were the length of ICU stay and in-hospital mortality. We grouped the patients into two, based on the time from onset of injury to surgery, an early group (within 24 h) and a late group (from 25 h to 7 days). Next, we performed multivariable analyses for analyzing the relevance between the timing of surgery and the length of ICU stay after adjusting for baseline characteristics using propensity score. We also performed the Cox survival analyses to evaluate in-hospital mortality.

**Results:**

From 236,698 trauma patients registered in JTDB, we analyzed 514 patients. The early group comprised 291 patients (56.6%), and the late group comprised 223 (43.4%). The length of ICU stay did not differ between the two groups (early, 10 days; late, 11 days; *p* = 0.29). There was no significant difference for length of ICU stay between the early and late group even after adjustment by multivariate analysis (*p* = 0.64). There was no significant difference in in-hospital mortality between the two groups (the early group 3.8%, the late group 2.2%, *p* = 0.32), and no significant difference was found in the Cox survival analysis.

**Conclusions:**

Our study showed that neither the length of ICU stay nor in-hospital mortality after spinal column stabilization or spinal cord decompression for cervical SCI significantly differed according to the timing of surgery between 24 h and 7 days.

## Background

Spinal cord injury (SCI) causes several negative outcomes, including physical, mental, economical, and social impairments that affect not only the patient, but also their family and caregivers. The number of patients is increasing yearly, and many studies regarding its treatment have been reported over the last several decades. Nevertheless, there is no recommended drug therapy for acute SCI [1, 2], and stabilization and decompression were only recommended for acute SCI in guidelines published in 2013 [3].

There are two phases following acute SCI: primary mechanical injury and secondary cellular injury [4, 5]. Primary mechanical injury is an irreversible damage caused by initial rapid spinal cord compression and trauma induced by a fracture or shearing force. Following primary injury, hemorrhage, vasospasm, ischemia, edema, excitotoxicity, inflammation, and apoptosis may bring about secondary cellular injury several hours after trauma, worsening the damage. The current management of SCI involves surgical intervention aimed at preventing and reducing the secondary damage [6].

Many reports comparing the neurological outcomes and mortality between cervical SCI patients who receive early surgery (within 24 h or 72 h) and those who receive late surgery have been published in the last decade. There are few large studies about the results of surgery in the early phase of cervical SCI. The results of previous cohort studies are conflicting. Some studies reported that early surgical intervention resulted in good neurological outcomes; however, other studies have suggested that surgery in the early phase worsened neurological outcomes and survival rates [7–13]. Given this, the optimal timing of surgical treatment remains unclear. Moreover, in some countries including Japan, on the one hand, it may be sometimes difficult to perform an operation within 24 h after injury in clinical practice, because it requires preparation of an operation room, anesthesiologists, or orthopedists, while on the other hand, reduction of prolonged intensive care unit (ICU) stay also has strong clinical implication [14, 15].

The aim of this study was to compare the length of the ICU along with in-hospital mortality among patients who had stabilization and/or decompression surgery for isolated cervical SCI within 24 h and that of within 7 days after trauma, using a Japanese nationwide trauma database.

## Methods

### Study design and setting of the study

The institutional ethics committee of the hospital approved the present study. Our analysis did not include personal identifying information, so the requirement for written informed consent was waived.

This was a retrospective cohort study the using Japan Trauma Data Bank (JTDB) database which is a nationwide, multicenter database administered by The Japanese Association for the Surgery of Trauma and The Japanese Association for Acute Medicine [16]. This database contained trauma cases classified as Abbreviated Injury Score (AIS) grade 3 or greater, and 244 hospitals, which included tertiary care emergency hospital (e.g., level I trauma centers) and less specialized emergency care hospitals, participated in the database [16]. Patients with cervical SCI (especially isolated cervical SCI cases without multiple trauma) are transported to not only the tertiary trauma centers but also the less specialized hospitals in Japan. The patients were registered in the JTDB at the last hospital if they were transferred to another hospital. The database contained information regarding patients’ age, sex, vital signs on scene, vital signs in the emergency room, mechanism of injury, diagnosis, AIS score [17], Injury Severity Score (ISS) [18], medical cares, ward, length of ICU stay, and length of hospital stay and mortality. Diagnosis and coding of the AIS were performed by doctors in charge of each institution. This database followed the patients till they discharged of the hospitals, transferred to other hospitals, or died in the hospitals.

### Definition and patient selection

We used the information on trauma patients enrolled in the JTDB between 2004 and 2015. We defined cervical SCI and graded the severity of cervical SCI using the AIS 2005 update 2008 code. For cervical SCI, AIS scores were coded from 1 to 6; a bigger number indicated a more severe neurological status. AIS 6 meant currently untreatable [17].

Inclusion criteria were as follows: (1) adult (15 years and above) patients, (2) having blunt isolated cervical SCI, (3) having undergone surgery within 7 days after injury, and (4) being admitted to the ICU. The exclusion criteria were as follows: (1) patients coded AIS 6 (currently untreatable patients), (2) absence of information about mortality, and (3) having died within 7 days after admission. We excluded patients who died within 7 days to avoid immortal time bias [19].

We grouped the participants into two groups: the early group who underwent surgery within 24 h after injury, and the late group who underwent surgery between 25 and 168 h (7 days) after injury. There is no standard definition for “early” surgical intervention in the literature regarding surgery for SCI with the range of “early” surgery being from within 4 h of injury to within 72 h of injury. The more recent trend is to define surgery within 24 h of injury as “early” surgery, and therefore, we defined early surgery as decompression or stabilization within 24 h of injury in the present study [7, 20, 21].

### Outcome measures

The primary outcome was the length of ICU stay. The secondary endpoint was in-hospital mortality.

### Statistical analysis

We grouped cervical cord injury patients who underwent surgical management into two groups: the early group and the late group according to timing of surgery. To compare the two groups, we compared categorical variables of the patients’ baseline characteristics using a chi-square or Fisher’s test. We analyzed continuous variables using the Student *t* test or Mann–Whitney *U* test, appropriately. We further performed a sub-group analysis and evaluated the relationship between the timing of surgery (day 1 to 7) and the outcomes, using Mantel–Haenszel trend test. We then performed multiple imputation, by which each missing value was replaced with a set of five substitute plausible values, to decrease the bias caused by incomplete data. A multivariable regression model was constructed for each imputed data set, and a single model was created by statistical inference about the results of the five imputed datasets [22, 23]. Next, the propensity score was estimated using a logistic regression model adjusted for the patient characteristics, cause of injury, and vital signs at the scene and on admission, as these variables have been shown to be prognostic predictors in previous studies (age, gender, prehospital vital signs, vital signs on hospital arrival, body temperature on arrival, GCS on arrival, ISS, and cervical AIS) [16, 21, 24]. We then performed a linear regression analysis for the length of the ICU stay for cervical SCI to compare different surgery timings. Lastly, we used the Cox proportional hazard regression analysis with variables that were the timing of operation (24 h vs. 7 days), the aim of operation (stabilization and decompression), and the propensity score to evaluate the secondary outcome, in-hospital mortality. The statistical significance threshold was a *p* value of less than 0.05. All analyses were carried out using SPSS software (IBM Corp., Armork, NY, USA, version 23).

## Results

**Fig. 1:**
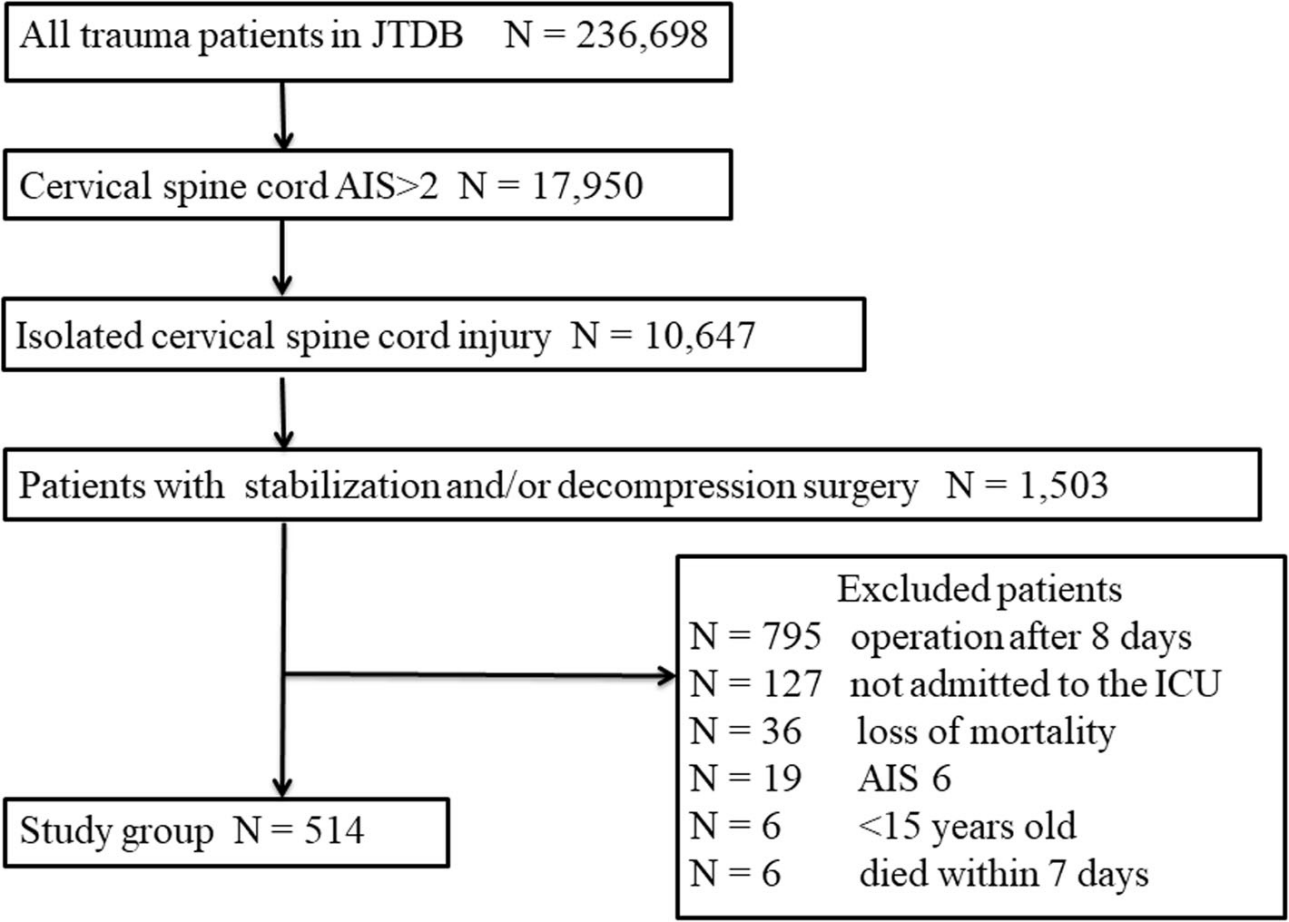
Flow diagram for patient selection

From a total of 236,698 trauma patients registered in the JTDB, we selected 514 patients who were diagnosed with isolated cervical SCI and underwent surgical treatment within 7 days (Fig. 1). Table 1 shows the patient demographic data in the early and late groups. Two hundred and ninety-one (56.6%) patients were operated on within less than 25 h after trauma. The ISS and AIS scores were significantly different in the two groups (*p* < 0.01 and *p* < 0.01); however, there was no significant difference in other variables. The procedures and outcomes are demonstrated in Table 2. The median time from the occurrence of injury to the start of surgical management was 8 h in the early group and 75 h in the late group (*p* < 0.01). Regarding the aims of surgery, stabilization tended to be a more frequent aim in the late group (*p* < 0.01). There was no significant difference in the length of hospital stay between the early and late groups (47 days vs. 36 days, *p* = 0.32).

**Table 1:** Demographics and clinical characteristics

Variables	Early group (*n* = 291)	Late group (*n* = 223)	*p* value
Age, years	65.0 (51.3–73.0)	65.0 (53.3–76.0)	0.50
Male, sex	240/290 (82.8)	185/223 (83.0)	0.95
Prehospital sBP	123 (100–144)	128 (106–147)	0.75
Prehospital PR	74 (63–84)	72 (64–83)	0.97
Prehospital RR	18 (18–24)	20 (18–24)	0.33
sBP on arrival	124 (106–143)	132 (109–152)	0.04
PR on arrival	70 (60–82)	70 (62–83)	0.88
RR on arrival	20 (16–22)	20 (16–23)	0.43
Body temperature on arrival	36.1 (35.4–36.7)	36.3 (35.6–36.8)	0.84
GCS on arrival	15 (14–15)	15 (14–15)	0.54
Injury Severity Score	16.5 (16.0–25.0)	16.0 (14.0–21.8)	< 0.01
Cervical Abbreviated Injury Score			< 0.01
2	14/291 (4.8)	15/223 (6.7)	
3	46/291 (15.8)	57/223 (25.6)	
4	124/291 (42.6)	103/223 (46.2)	
5	107/291 (36.8)	48/223 (21.5)	
Cause of injury			0.96
Traffic accident	72/290 (24.8)	56/221 (25.3)	
Fall	189/290 (65.2)	146/221 (66.1)	
Sports	12/290 (4.1)	8/221 (3.6)	
Others	17/290 (5.9)	11/221 (5.0)	

Data given as number of positive observations/total number of observations (percentage) or as median (interquartile range). For each variable, the number of missing observations can be obtained as the difference between the total number of patients in each phase and the total number of observations. *sBP* systolic blood pressure, *PR* pulse rate, *RR* respiratory rate, *GCS* Glasgow Coma Scale

**Table 2:** Procedures, ICU periods, hospital periods, and mortality

Variables	Early group (*n* = 291)	Late group (*n* = 223)	*p* value
Traction	23/291 (7.9)	24/223 (10.8)	0.27
Aim for Surgery			< 0.01
Stabilization	169/291 (58.1)	164/223 (74.5)	
Decompression	56/291 (19.2)	35/223 (15.7)	
Stabilization and decompression	52/291 (17.9)	20/223 (9.0)	
Others	14/291 (4.8)	4/223 (1.8)	
Time from injury to surgery, hours	8 (5–13)	75 (42–111)	< 0.01
Length of ICU stay, days	10 (3–39)	11 (4–28)	0.87
Length of hospital stay, days	48 (27–66)	34 (21–57)	0.29
In-hospital mortality	11/291 (3.8)	5/223 (2.2)	0.32

Data given as number of positive observations/total number of observations (percentage) or as median (interquartile range). For each variable, the number of missing observations can be obtained as the difference between the total number of patients in each phase and the total number of observations. *ICU* intensive care unit

**Fig. 2:**
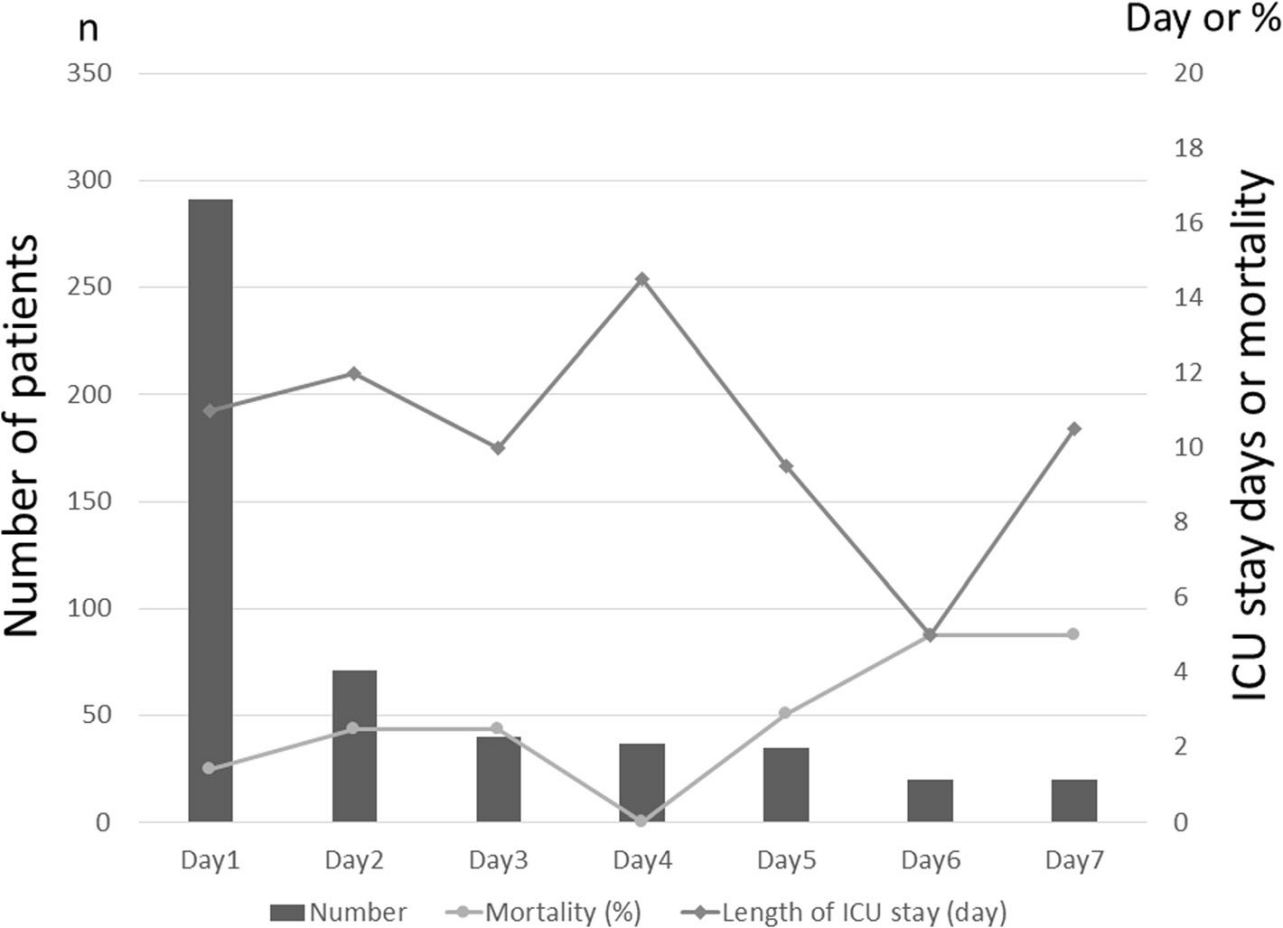
Number of patients, in-hospital mortality, and length of ICU stay for each of the 7 days after injury

For the primary outcome, the median length of ICU stay was 10 days in the early group and 11 days in the late group (*p* = 0.87). There were no significant differences in the length of ICU stay among 7 days as presented in Fig. 2 (Mantel–Haenszel trend test, *p* = 0.79). There was no significant difference for the length of ICU stay between the early and late group even after adjustment for the timing of surgery, the aim of surgery (stabilization and decompression), and the propensity score calculated by variables about patient characteristics using multivariate analysis (*p* = 0.64).

**Fig. 3:**
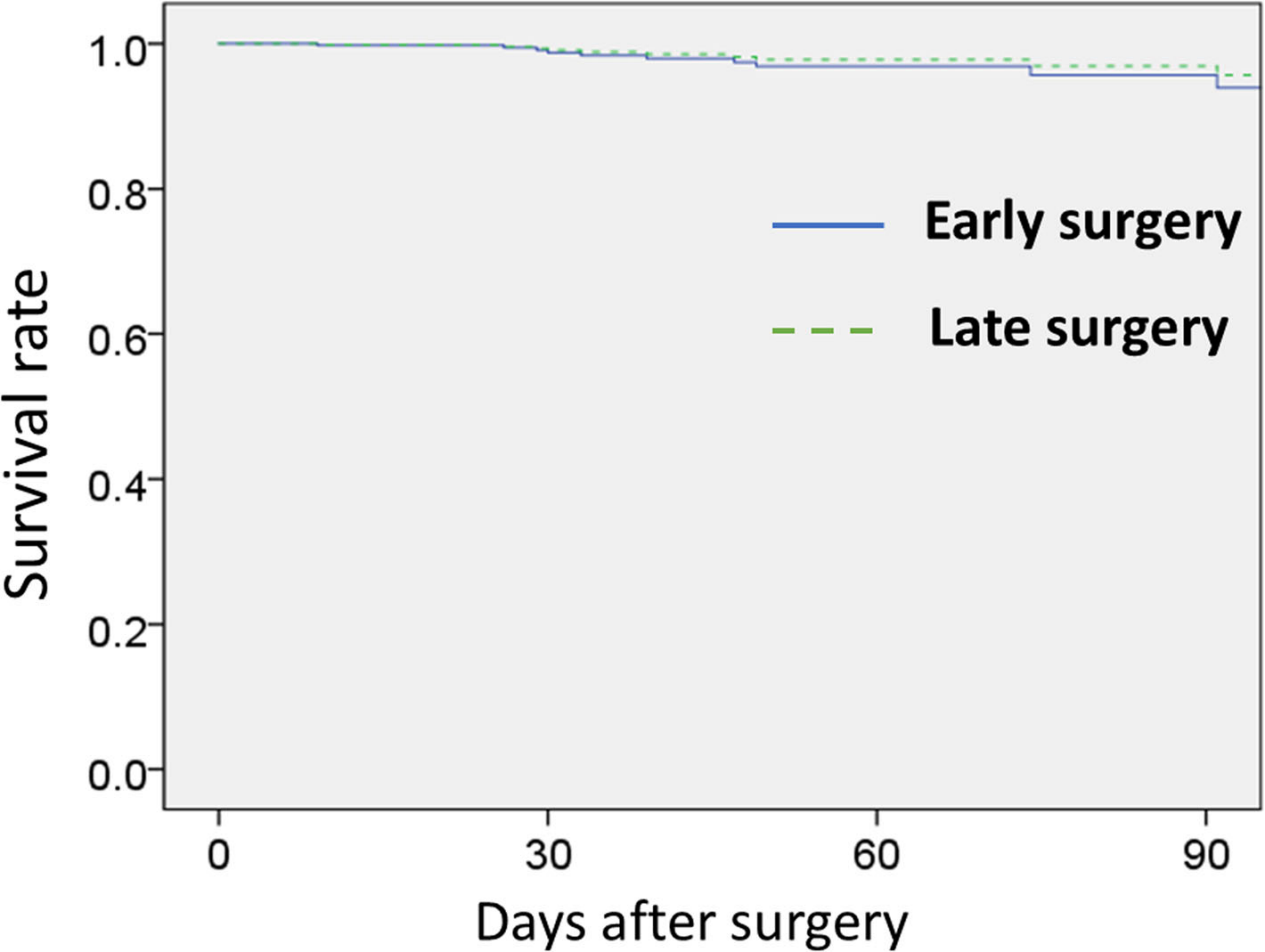
Survival curve between early and late surgery after operation. Early surgery, operation performed within 24 h; late surgery, operation performed from 25 h to 7 days. The propensity score adjusted Cox regression analysis found no significant difference between early and late operation for the in-hospital mortality of cervical spinal cord injury (*p* = 0.53)

For the secondary outcome, there was no significant difference in in-hospital mortality between the two groups (the early group 3.8%, the late group 2.2%, *p* = 0.32). There were no significant differences in the in-hospital mortality among 7 days (Mantel–Haenszel trend test, *p* = 0.84). As shown in Fig. 3, the Cox regression analysis found no significant difference between the early and late operation for the in-hospital mortality of cervical SCI (*p* = 0.53)

## Discussion

In the current study, we analyzed 514 isolated cervical SCI cases who received surgery within 1 week after trauma in Japanese nationwide trauma database. We found that neither the length of ICU stay nor the in-hospital mortality after spinal column stabilization or spinal cord decompression for cervical SCI significantly differed according to the timing of surgery between 24 h and 7 days.

The strength of our study was that we included a large study population from 244 institutions. Previous reports on the timing of surgery for SCI included much smaller sample sizes and/or were not multicenter studies, especially previous reports regarding cervical SCI. Table 1 shows that more patients were classified as AIS 5 in the early group compared to the late group. Furthermore, ISS scores tended to be higher in the early group. The severity of trauma was worse in the early group; however, other characteristics, such as age, vital signs on arrival, and cause of injury, were similar among the two groups. We adjusted these measured confounding factors using propensity score and multivariable analyses to evaluate the outcomes in our study. Interestingly, although the injury to surgery time was longer in the late group (by definition), there was no significant difference in ICU period as well as hospital stay period in this cohort. We also found that in-hospital mortality after spinal column stabilization and/or spinal cord decompression for cervical SCI did not significantly differ according to the timing of surgery between 24 h and 7 days. Although further prospective studies are required for confirmation, these findings may help to work out a strategy to manage SCI patients in the future.

Cervical SCI sometimes can be a fatal injury and need intensive care in the acute phase, so we set the main outcome to be in the length of ICU stay; however, there are few studies focused on ICU period in SCI patients after operative management. Most of the previous studies on SCI included not only cervical SCI, but also thoracic and lumbar SCI, which are generally not lethal injuries. Low mortality rates were reported, and the length of hospital stay and complications (for instance, pulmonary embolism and pneumoniae) were considered as the main benchmarks of the safety and non-neurological outcomes after early and late surgery in SCI patients in these papers, and the results are conflicting [7, 11, 13, 25]. As for cervical SCI, results on mortality and other variables in early and late surgery patients are also controversial [7, 9, 10, 12, 13, 26]. Our large multicenter study showed no difference in the length of ICU stay and mortality rates in the early and late surgery groups, so our results may help to resolve this confusing situation. If the timing of surgery is not associated with ICU days, which is one of the big issues for cervical SCI patients, we will have more choice of clinical treatment. If we do not perform surgery within 24 h due to some reasons (for example, no orthopedic specialist available or the patient not being in a suitable condition for general anesthesia), we can prepare for surgery or transport the patient to a specialized hospital, even after 24 h.

### Limitations

There were some limitations in our study. First, the JTDB does not contain information about neurological status before and after surgery. For example, the database did not include Frankel Grade or American Spinal Injury Association Impairment Scale, so we could not evaluate the relevance of the timing of surgery for cervical SCI and neurological improvements, that is, the efficacy of surgery for SCI. We did assess the severity of cervical SCI using the AIS; however, many previous studies have used the levels of injury, in terms of AO Spine Trauma Classification or Frankel Grade. Therefore, we could not directly compare the present study with many previous studies. Second, we could not obtain information about the indication for surgery, the reason behind the timing of surgery, the comorbidities of the patients, the complications of the operation, or the cause of death. Consequently, we could not analyze the direct effect of timing of surgery on the length of the ICU stay. Third, this database does not include information about comorbidities, number of days on ventilator, complications, and neurological status; therefore, whether there were some causes that had made the hospital stay longer was not clear in our study. Fourth, regarding the AIS coding rule about the spine, paralysis should be coded according to its status at 24 h post-injury [17]. It may be possible that the current study included the patients whose AIS could have changed within 24 h as a result of operation itself or in the natural course (i.e., unrelated to operation) in the early group.

## Conclusion

Our study suggests that even if the injury to surgery time was longer, there was no significant difference in the ICU period as well as hospital stay period for cervical SCI patients who had undergone early and late surgical management, when it was performed within 1 week of trauma. We also found that there was no significant difference in in-hospital mortality between early and late procedure for cervical SCI. Although further prospective studies are required for confirmation, these findings may help to work out a strategy to manage SCI patients.

## Data Availability

The datasets used and/or analyzed during the current study are available from the corresponding author on reasonable request. Please contact corresponding author for data requests.

## Abbreviations

AIS: Abbreviated Injury Score
ISS: Injury Severity Score
JTDB: Japan Trauma Data Bank
SCI: Spinal cord injury
